# Depletion of runt-related transcription factor 2 (RUNX2) enhances SAHA sensitivity of *p53*-mutated pancreatic cancer cells through the regulation of mutant p53 and TAp63

**DOI:** 10.1371/journal.pone.0179884

**Published:** 2017-07-03

**Authors:** Takehiro Ogata, Mizuyo Nakamura, Meijie Sang, Hiroyuki Yoda, Kiriko Hiraoka, Danjing Yin, Mexiang Sang, Osamu Shimozato, Toshinori Ozaki

**Affiliations:** 1 Laboratory of DNA Damage Signaling, Chiba Cancer Center Research Institute, Chiba, Japan; 2 Department of Regenerative Medicine, Graduate School of Medicine, University of Toyama, Toyama, Japan; 3 Laboratory of Cancer Genetics, Chiba Cancer Center Research Institute, Chiba, Japan; 4 Research Center, Fourth Hospital of Hebei Medical University, Shijiazhuang, Hebei, P.R. China; German Cancer Research Center, GERMANY

## Abstract

Suberoylanilide hydroxamic acid (SAHA) represents one of the new class of anti-cancer drugs. However, multiple lines of clinical evidence indicate that SAHA might be sometimes ineffective on certain solid tumors including pancreatic cancer. In this study, we have found for the first time that RUNX2/mutant p53/TAp63-regulatory axis has a pivotal role in the determination of SAHA sensitivity of *p53*-mutated pancreatic cancer MiaPaCa-2 cells. According to our present results, MiaPaCa-2 cells responded poorly to SAHA. Forced depletion of mutant *p53* stimulated SAHA-mediated cell death of MiaPaCa-2 cells, which was accomapanied by a further accumulation of γH2AX and cleaved PARP. Under these experimental conditions, pro-oncogenic RUNX2 was strongly down-regulated in mutant *p53*-depleted MiaPaCa-2 cells. Surprisingly, *RUNX2* silencing augmented SAHA-dependent cell death of MiaPaCa-2 cells and caused a significant reduction of mutant p53. Consistent with these observations, overexpression of RUNX2 in MiaPaCa-2 cells restored SAHA-mediated decrease in cell viability and increased the amount of mutant p53. Thus, it is suggestive that there exists a positive auto-regulatory loop between RUNX2 and mutant p53, which might amplify their pro-oncogenic signals. Intriguingly, knockdown of mutant *p53* or *RUNX2* potentiated SAHA-induced up-regulation of TAp63. Indeed, SAHA-stimulated cell death of MiaPaCa-2 cells was partially attenuated by *p63* depletion. Collectively, our present observations strongly suggest that RUNX2/mutant p53/TAp63-regulatory axis is one of the key determinants of SAHA sensitivity of *p53*-mutated pancreatic cancer cells.

## Introduction

The overall survival rate of the patients with various solid tumors has been clearly prolonged due to the improved therapy and surgical procedures. Among them, however, pancreatic cancer remains the most lethal malignant tumor with an extremely poor prognosis (overall survival rate of around 7%) in spite of the extensive efforts [1–4]. Indeed, less than 10% of the patients with pancreatic cancer are diagnosed at an early stage due to the difficulty in its early detection, and therefore most of the remaining patients do not have a chance to take a surgical resection attributed to its late stage. Unfortunately, majority of the patients who have received the surgery, suffer recurrence [5]. These advanced cases exhibit a severe resistance to the existing therapeutic modalities.

It has been reported that *p53* (~75%), *KRAS* (>90%), *CDKN2A/p16* (>90%) and *SMAD4/DPC4* (~50%) are frequently mutated in pancreatic cancer, and these mutations are tightly linked to its malignant behavior [6]. p53 is a representative tumor suppressor with a sequence-specific transactivation potential. Upon DNA damage, p53 quickly becomes stabilized and then transactivates its target genes implicated in the induction of cell cycle arrest, cellular senescence and/or cell death. While, *p53* is frequently mutated in human tumor tissues (nearly 50% of tumors) and over 90% of its mutations occur within the genomic region encoding its sequence-specific DNA-binding domain. Therefore, mutant p53 lacks its sequence-specific transactivation ability as well as pro-apoptotic function (loss of function), and sometimes acquires pro-oncogenic property (gain of function). Importantly, mutant p53 acts as a dominant-negative inhibitor against wild-type p53 and contributes to the acquisition and/or maintenance of a drug-resistant phenotype of advanced tumors [7, 8]. In fact, certain tumor cells bearing *p53* mutations display a serious drug-resistant phenotype [9–11].

Meanwhile, p53 is a founding member of a small tumor suppressor p53 family composed of p53, p73 and p63 [12]. *p73/p63* encodes a transcription-competent TA and a transcription-deficient ΔN isoform arising from an alternative splicing and an alternative promoter usage, respectively. As expected from their structural similarities to p53, TA isoforms are capable to transactivate the overlapping set of p53-target genes involved in the promotion of cell cycle arrest, cellular senescence and/or cell death. Similar to mutant p53, NH_2_-terminally-truncated ΔNp73/ΔNp63 with pro-oncogenic potential exhibits a dominant-negative behaviour against TAp73/TAp63. Like p53, TAp73/TAp63 is induced in response to DNA damage such as anti-cancer drug treatment and then exerts its pro-apoptotic function to eliminate tumor cells [12]. In a sharp contrast to *p53*, *p73* and *p63* are rarely mutated in human primary tumor tissues [13]. Therefore, *p73* and *p63* are expressed as wild-type forms both in tumor tissues and their corresponding normal ones. Notably, it has been demonstrated that TAp73/TAp63 is required for p53-dependent cell death in response to DNA damage, whereas TAp73/TAp63 has an ability to promote DNA damage-mediated cell death in the absence of functional p53 [14].

RUNX2, runt-related transcription factor 2, is a nuclear sequence-specific transcription factor essential for osteoblast differentiation and bone formation [15, 16]. In addition to its pro-osteogenic function, the possible contribution of RUNX2 to tumorigenesis and/or metastasis has been increasingly recognized. For example, *RUNX2* is aberrantly overexpressed in a variety of tumors such as breast cancer, prostate cancer, pancreatic cancer, gastric cancer and melanoma [17–20]. RUNX2 transactivates its direct target genes implicated in angiogenesis, invasiveness and metastasis including *Vegf*, *Spp1*, *MMP9* and *MMP13* [21, 22]. Although gemcitabine (GEM) is the present gold standard of anti-cancer drug for the treatment of pancreatic cancer patients, its efficacy is quite limited due to the inherited or the acquired drug-resistant phenotype of pancreatic cancer [23]. Recently, we have found for the first time that RUNX2 attenuates p53 family-dependent cell death following DNA damage, and *RUNX2* gene silencing mediated by siRNA clearly enhances GEM sensitivity of pancreatic cancer cells irrespective of their *p53* status [24–27].

Histone deacetylases (HDACs) are a family of enzymes which catalyze the hydrolytic release of acetyl groups from lysine residues of their target proteins. It has been well documented that HDACs play a crucial role in the modulation of a broad range of biological processes including cell cycle progression, cell death, stress response and differentiation through the regulation of their target gene transcription [28, 29]. Of note, an emerging evidence strongly indicates the potential role of HDACs in human diseases. For instance, it has been described that a higher expression level of HDAC2 is required for the maintenance of malignant phenotypes of colon cancer cells [30]. Lee *et al*. demonstrated that HDAC6 is responsible for oncogene-mediated tumorigenesis in mice [31]. Intriguingly, Stojanovic *et al*. found that HDAC1 and HDAC2 contribute to the maintenance of a higher expression level of mutant p53 in pancreatic cancer cells [32]. With these in mind, HDAC inhibitors (HDACIs) have been considered to be the potential anti-cancer drugs, and currently under intense investigation. Among these candidates, suberoylanilide hydroxamic acid (SAHA) has been approved by the Food and Drug Administration for the treatment of T cell lymphoma patients [33, 34]. Theoretically, SAHA facilitates the accumulation of acetylated cellular proteins such as histones and transcription factors, and thus induces the dynamic changes of gene expression in tumor cells. Unfortunately, the extensive clinical trials suggest that SAHA might be sometimes ineffective on certain solid tumors [35].

To overcome this serious burden, it is quite important to clarify the precise molecular basis of this SAHA-resistant phenotype of the advanced solid tumors. In the present study, we have focused on *p53*-mutated pancreatic cancer cells, and found that RUNX2/mutant p53/TAp63-regulatory axis plays a pivotal role in the modulation of SAHA-mediated cell death.

## Materials and methods

### Cell culture and transfection

Human pancreatic cancer MiaPaCa-2 and Panc-1 cells with *p53* mutation were purchased from ATCC (American Type Culture Collection), and maintained in Dulbecco’s Modified Eagle’s Medium (DMEM) supplemented with heat-inactivated 10% fetal bovine serum (Invitrogen, Carlsbad, CA, USA) and 50 units/ml of penicillin/streptmycin. *p53*-proficient human breast cancer MCF-7 cells were caultured in RPMI-1640 medium containing heat-inactivated 10% fetal bovine serum and 50 units/ml of penicillin/streptmycin. Cells were cultured in incubators with humidified atmospheres of 5% CO_2_ and 95% air at 37°C. For transfection, cells were transfected with the indicated expression plasmids using LipofectAmine 2000 according to the manufacturer’s instructions (Invitrogen).

### RNA preparation and RT-PCR

Cells were treated with the indicated concentrations of SAHA. At the indicated time periods after treatment, total RNA was purified using RNeasy Mini Kit according to the manufacturer’s suggestions (Qiagen, Hilden, Germany). One microgram of total RNA was reverse-transcribed by using SuperSprict VILO cDNA synthesis system following the manufacturer’s protocols (Invitrogen). After PCR reaction, the resultant products were resolved in 1.5% agarose gel electrophoresis and visualized by ethidium bromide staining. Gene expression was normalized relative to that of the housekeeping gene *GAPDH*. The oligonucleotide primers used for PCR-based amplification were as follows: *p53*, 5’-CTGCCCTCAACAAGATGTTTTG-3’ (forward) and 5’-CTATCTGAGCAGCGCTCATGG-3’ (reverse); *TAp63*, 5’-GACCTGAGTGACCCCATGTG-3’ (forward) and 5’-CGGGTGATGGAGAGAGAGCA-3’ (reverse); *TAp73*, 5’- TCTGGAACCAGACAGCACCT-3’ (forward) and 5’- GTGCTGGACTGCTGGAAAGT-*3*’ (reverse); *RUNX2*, 5’-TCTGGCCTTCCACTCTCAGT-3’ (forward) and 5’-GACTGGCGGGGTGTAAGTAA-3’ (reverse); *p21*^*WAF1*^, 5’-ATGAAATTCACCCCCTTTCC-3’ (forward) and 5’-CCCTAGGCTGTGCTCACTTC-3’ (reverse); *NOXA*, 5’-CTGGAAGTCGAGTGTGCTACT-3’ (forward) and 5’-TCAGGTTCCTGAGCAGAAGAG-3’ (reverse); *BAX*, 5’-AGAGGATGATTGCCGCCGT-3’ (forward) and 5’-CAACCACCCTGGTCTTGGAT-3’ (reverse); *GAPDH*, 5’-ACCTGACCTGCCGTCTAGAA-3’ (forward) and 5’-TCCACCACCCTGTTGCTGTA-3’ (reverse).

### Western blot analysis

Cells were washed in ice-cold 1 x PBS (phosphate-buffered saline) and lysed in lysis buffer containing 25 mM Tris-HCl, pH 8.0, 137 mM NaCl, 2.7 mM KCl, and 1% Triton X-100 supplemented with a commercial protease inhibitor mixture (Sigma, St. Louis, MO, USA). Equivalent amounts of protein (50 μg) were separated on 10% SDS-polyacrylamide gel electrophoresis and then electro-transferred onto a polyvinylidene difluoride membrane (Immobilon; Merck Millipore, Amsterdam, Netherlands). The membrane was probed with mouse monoclonal anti-p53 (DO-1; Santa Cruz Biotechnology, Santa Cruz, CA, USA), rabbit polyclonal anti-TAp73 (GeneTex, Irvine, CA, USA), rabbit polyclonal anti-TAp63 (GeneTex), rabbit polyclonal anti-p21^WAF1^ (Cell Signaling Technologies, Beverly, MA, USA), rabbit polyclonal anti-BAX (Cell Signaling Technologies), rabbit polyclonal anti-RUNX2 (Cell Signaling Technologies), rabbit polyclonal anti-PARP (Cell Signaling Technologies), mouse monoclonal anti-γH2AX (2F3; BioLegend, San Diego, CA, USA) or with rabbit polyclonal anti-actin antibody (20–33, Sigma) at room temperature for 1 h. After extensive washing in Tris-buffered saline containing 0.1% Tween 20 (TBS-T), the membrane was incubated with horseradish peroxidase-conjugated goat anti-mouse or anti-rabbit IgG (Invitrogen) at room temperature for 1 h. Visualization of horseradish peroxidase was achieved by using an enhanced chemiluminescence detection system (ECL; GE Healthcare Life Science, Piscataway, NJ, USA).

### Indirect immunofluorescence

Cells were treated with DMSO, the indicated concentrations of SAHA or left untreated. Forty-eight hours after treatment, cells were fixed in 3.7% formaldehyde at room temperature for 30 min, treated with 0.1% Triton X-100 in 1 x PBS at room temperature for 5 min, and blocked with 3% BSA in 1 x PBS at room temperature for 1 h. After washing in 1 x PBS, cells were incubated with anti-γH2AX, anti-p63 or with anti-p53 antibody at room temperature for 1 h. After wash in 1 x PBS, cells were incubated with FITC-conjugated anti-mouse IgG at room temperature for 1 h. After washing in 1 x PBS, cell nuclei were stained with DAPI (Vector Laboratories, Peterborough, UK). Fluorescent images were captured using a confocal microscope.

### siRNA-mediated knockdown

Cells were transfected with control scrambled siRNAs (Santa Cruz Biotechnology), *RUNX2* siRNA (Dharmacon, Loughborough, UK), *p63* siRNA (Santa Cruz Biotechnology) or with *p53* siRNA (Santa Cruz Biotechnology) using LipofectAmine 2000 (Invtrogen). The final concentration of each siRNA was 10 nM. Silencing of the indicated genes was evaluated by immunoblotting and RT-PCR.

### Cell survival assay

Cells were seeded into 96-well plate at a concentration of 1.5 x 10^3^ cells/well, and allowed to attach overnight. Cells were then treated with DMSO, the indicated concentrations of SAHA or left untreated. At the indicated time points post-treatment, their proliferation was measured by Cell Counting kit-8 reagent according to the manufacturer’s instructions (Dojindo Molecular Technologies, Rockville, MD, USA). Experiments were performed in triplicate.

### Flow cytometry

The standard protocol for propidium iodide (PI) staining was employed in the present flow cytometric analysis. In brief, floating as well as adherent cells were harvested, and fixed in ice-cold 70% ethanol. Following fixation, cells were treated with 1 μg/ml of PI and 1 μg/ml of RNase A at 37°C for 30 min. After the incubation, cells were sorted on the basis of their DNA content by flow cytometry (FACS Calibur, BD Biosciences, Franklin Lakes, NJ, USA).

### Statistical analysis

Results were shown as mean ± S.D. Student's *t*-test was used to assess differences among groups. *p* value of < 0.05 was considered as statistically significant.

## Results

### Poor response of *p53*-mutated human pancreatic cancer MiaPaCa-2 cells to SAHA

**Fig 1:**
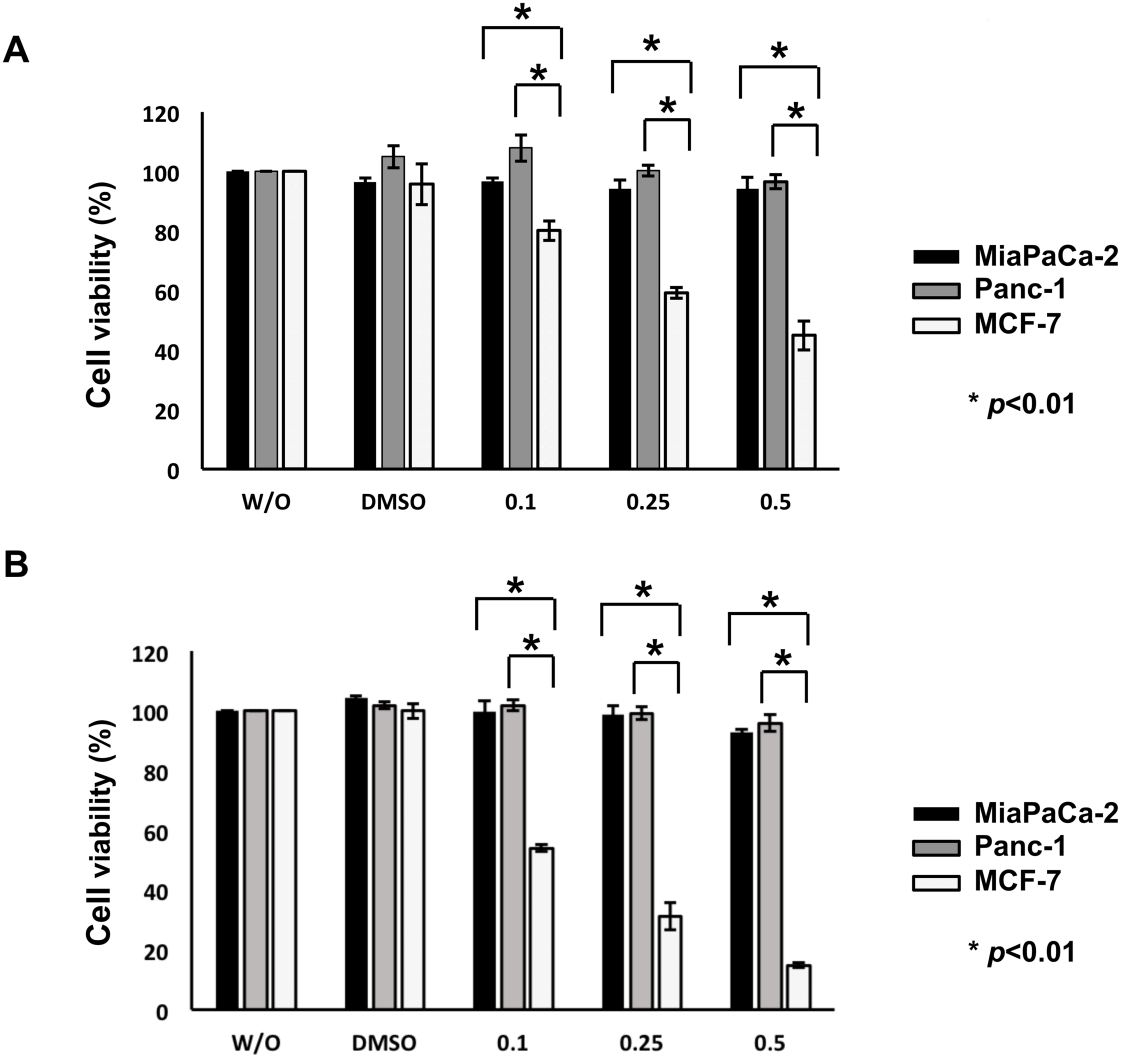
*p53*-mutated human pancreatic cancer MiaPaCa-2 and Panc-1 cells respond poorly to SAHA. MiaPaCa-2 (solid boxes), Panc-1 (grey boxes) and *p53*-proficient human breast cancer MCF-7 (open boxes) cells were exposed to DMSO, the indicated concentrations of SAHA or left untreated (W/O). Twenty-four (A) and 48 h (B) after treatment, cell viability was examined by standard WST cell survival assay.

To ask whether histone deacetylase inhibitor SAHA could efficiently induce cell death of pancreatic cancer cells bearing *p53* mutation, MiaPaCa-2 and Panc-1 cells were treated with DMSO or with the increasing concentrations of SAHA. *p53*-proficient human breast cancer MCF-7 cells which have been shown to be highly sensitive to SAHA [36], were employed as a positive control, and Panc-1 cells have been demonstrated to be highly resistant to SAHA [37]. Twenty-four and 48 h after treament, cell viability was examined by standard WST cell survival assay. As clearly shown in Fig 1, cell viability of MCF-7 cells was sharply decreased in response to SAHA in a dose-dependent manner, whereas SAHA had a merginal effect on MiaPaCa-2 and Panc-1 cells. FACS analysis revealed that at most 10% of MiaPaCa-2 and Panc-1 cells carry sub-G1 DNA content following 48 h of SAHA exposure (0.5 μM) (S1 Fig). Therefore, it is conceivable that, like Panc-1 cells, MiaPaCa-2 cells poorly respond to SAHA, and we then focused on MiaPaCa-2 cells for further study.

### Inverse relationship between the expression levels of mutant p53/RUNX2 and TAp63 in response to SAHA

**Fig 2:**
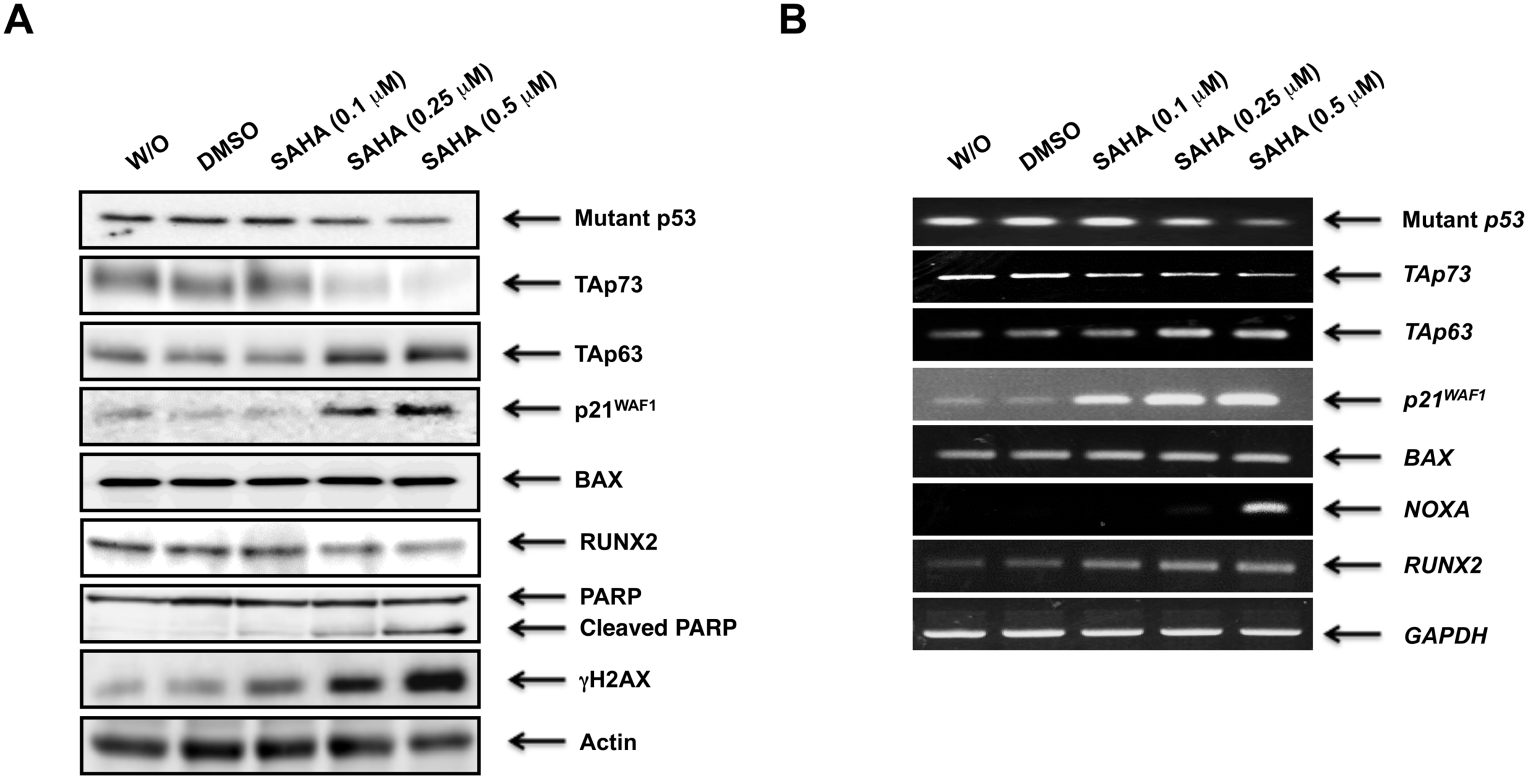
Inverse relationship between the expression levels of mutant p53/RUNX2 and TAp63 in MiaPaCa-2 cells following SAHA exposure. MiaPaCa-2 cells were treated with DMSO, the increasing amounts of SAHA or left untreated. Forty-eight hours after treatment, cell lysates and total RNA were prepared and subjected to immunoblotting (A) and RT-PCR (B), respectively. Actin and *GAPDH* were used as a loading and an internal control, respectively.

**Fig 3:**
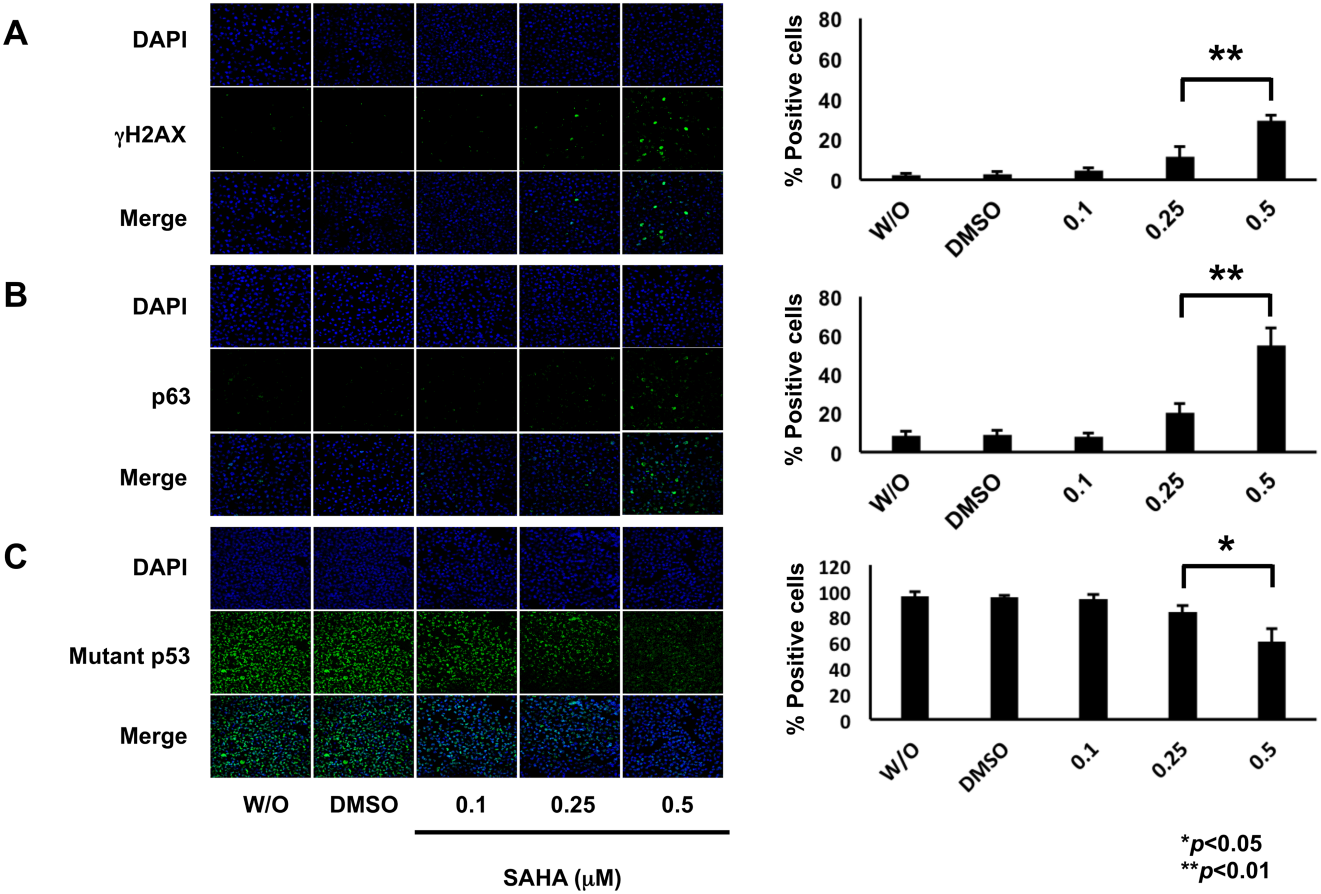
SAHA-dependent induction of γH2AX as well as p63 and reduction of mutant p53. MiaPaCa-2 cells were treated with DMSO, the indicated concentrations of SAHA or left untreated. Forty-eight hours after treatment, cells were fixed and incubated with anti-γH2AX (A), anti-p63 (B) or with anti-p53 (C) antibody (green). Cell nuclei were stained with DAPI (blue) (left panels). Based on the results obtained from the immunostaing experiments, number of γH2AX-, p63- or mutant p53-positive cells was scored (right bar graphs).

To gain insight into better understanding of the precise molecular mechanism(s) behind a poor response to SAHA of MiaPaCa-2 cells, we sought to examine the expression patterns of pro-apoptotic p53 family members (mutant p53, TAp73 and TAp63) and their related gene products following SAHA exposure. As shown in Fig 2A, SAHA treatment resulted in a clear increase in the amount of γH2AX which is a reliable marker for double-strand breaks (DSBs) in DNA, implying that MiaPaCa-2 cells receive SAHA-mediated DNA damage. In accordance with the results obtained from flow cytometric analysis, SAHA-dependent proteolytic cleavage of PARP was detectable. Meanwhile, TAp63 and mutant p53 were up- and down-regulated upon SAHA treatment, respectively. In contrast to TAp63, another p53 family member TAp73 was markedly reduced in response to SAHA. For p53 family-target genes, the expression levels of *p21*^*WAF1*^ and *NOXA* were elevated following SAHA exposure (Fig 2B), which might be at least in part due to SAHA-induced up- and down-regulation of TAp63 and mutant p53, respectively. However, *BAX* remained unchanged regardless of SAHA treatment. The amount of RUNX2 was lowered at protein level in the presence of SAHA. To confirm the results obtained from immunoblotting, we performed indirect immunofluorescence staining. To this end, MiaPaCa-2 cells were exposed to DMSO, the increasing concentrations of SAHA or left untreated. Forty-eight hours after treatment, cells were fixed and incubated with anti-γH2AX, anti-p63 or with anti-p53 antibody. As shown in Fig 3, SAHA-mediated induction of γH2AX as well as p63, and reduction of mutant p53 were observed.

Similar to MiaPaCa-2 cells, SAHA-induced up- and down-regulation of TAp63 and mutant p53/TAp73/RUNX2 was detected in Panc-1 cells, respectively (S2 Fig). However, SAHA-dependent proteolytic cleavage of PARP was not seen in Panc-1 cells under our experimental conditions. Consistent with these results, Arnold *et al*. reported that Panc-1 cells show the poor response to SAHA without PARP cleavage [37]. Considering that the extent of SAHA-mediated up-regulation of TAp63 and its downstream target p21^WAF1^ in Panc-1 cells is smaller than that of MiaPaCa-2 cells, it is likely that SAHA-stimulated cell cycle arrest/cell cycle retardation takes place in Panc-1 cells, but Panc-1 cells undergo cell death to a lesser degree relative to MiaPaCa-2 cells. Indeed, SAHA-mediated increase in sub-G1 cell population of Panc-1 cells was smaller than that of MiaPaCa-2 cells (S1 Fig). Although p53 mutant types (R248W/R273H for MiaPaCa-2 cells and R273H for Panc-1 cells) might affect the results of SAHA exposure, the precise molecular basis of this phenomenon is elusive.

### Knockdown of mutant *p53* improves SAHA sensitivity in association with marked down- and up-regulation of RUNX2 and TAp63, respectively

**Fig 4:**
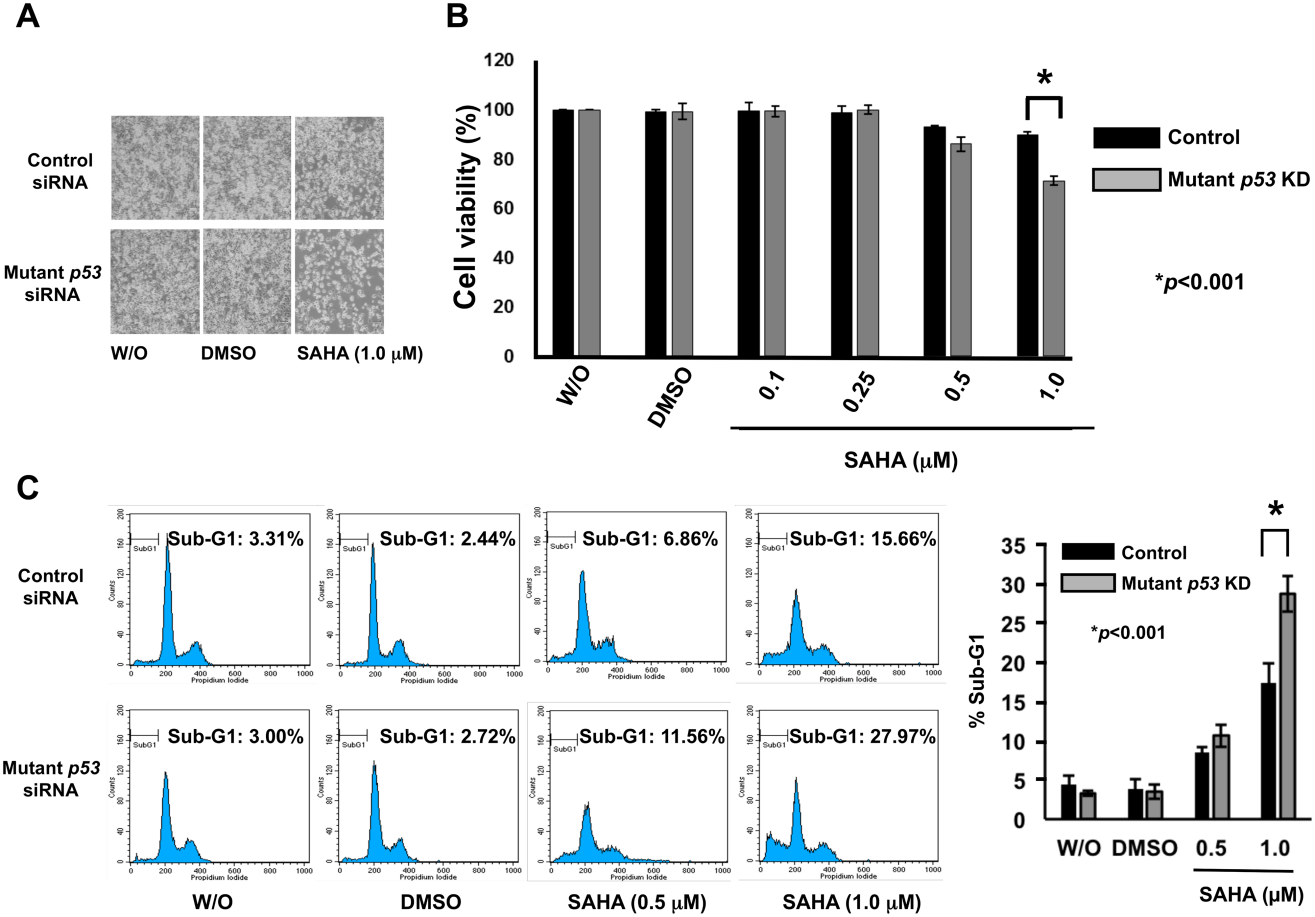
Silencing of mutant *p53* in MiaPaCa-2 cells stimulates SAHA-dependent decrease and increase in cell viability and cell death, respectively. (A) Phase-contrast micrographs. MiaPaCa-2 cells were transfected with control siRNA or with siRNA against *p53*, and then treated with DMSO, 1 μM of SAHA or left untreated. Forty-eight hours after treatment, the representative pictures were taken. (B) WST assay. MiaPaCa-2 cells were transfected with control siRNA or with siRNA against *p53*, and treated with DMSO or with the indicated concentrations of SAHA. Forty-eight hours after SAHA exposure, cells were analyzed by the standard WST cell survival assay. Solid and grey boxes indicate control siRNA- and *p53* siRNA-transfected cells, respectively. (C) FACS analysis. MiaPaCa-2 cells were transfected with control siRNA or with siRNA against *p53*, and treated with DMSO or with the indicated concentrations of SAHA. Forty-eight hours after treatment, floating and adherent cells were harvested and subjected to flow cytometric analysis. Solid and grey boxes indicate control siRNA- and *p53* siRNA-transfected cells, respectively.

Considering that mutant p53 contributes to the acquisition and/or maintenance of drug-resistant phenotype of certain malignant tumor cells [9–11], we asked whether forced depletion of mutant *p53* could enhance SAHA sensitivity of MiaPaCa-2 cells. Since MiaPaCa-2 cells do not carry wild-type *p53* allele, we have employed siRNA against *p53* (sc-29435, Santa Cruz Biotechnology) to deplete mutant *p53* in the present study. MiaPaCa-2 cells were transfected with control siRNA or with siRNA targeting *p53*, and then exposed to DMSO, 1 μM of SAHA or left untreated. As seen in Fig 4A, knockdown of mutant *p53* led to a remarkable decrease in number of attached cells following SAHA exposure as compared to SAHA-treated non-silencing control cells. Consistent with these observations, WST cell survival assays demonstrated that SAHA-dependent decline in cell viabilty is further stimulated by mutant *p53* knockdown (Fig 4B). Under the same experimental conditions, we have conducted flow cytometric analysis. As shown in Fig 4C, silencing of mutant *p53* increased the proportion of cells in sub-G1 phase in response to SAHA. Thus, it is likely that mutant *p53* knockdown improves SAHA sensitivity of MiaPaCa-2 cells.

**Fig 5:**
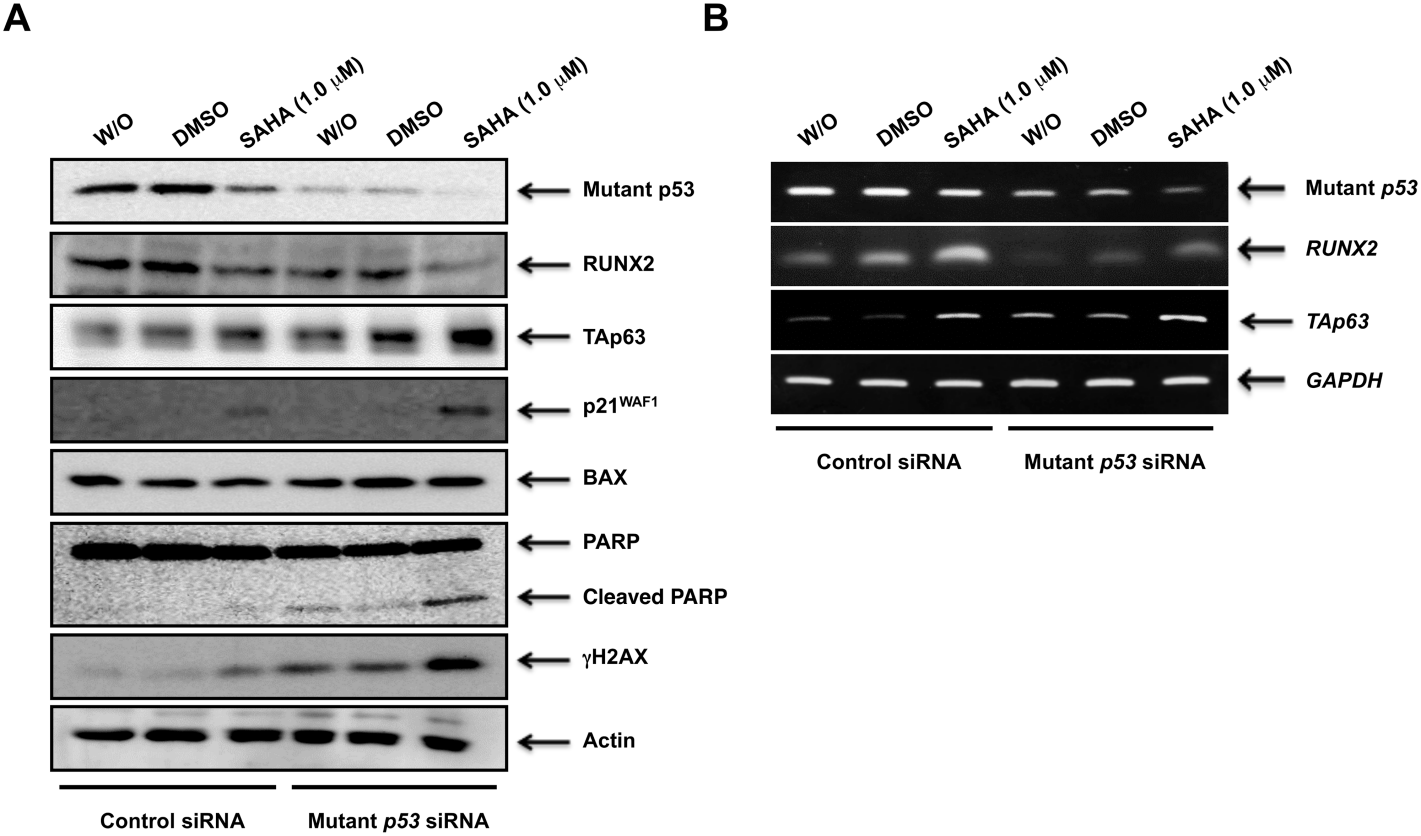
Forced depletion of mutant *p53* augments SAHA-mediated accumulation of TAp63 and reduction of RUNX2. MiaPaCa-2 cells were transfected and treated as in Fig 4A. Forty-eight hours post-treatment, cell lysates and total RNA were prepared and analyzed by immunoblotting (A) and RT-PCR (B), respectively. Actin and *GAPDH* were used as a loading and an internal control, respectively.

To understand the molecular mechanisms how mutant *p53* knockdown could enhance SAHA sensitivity of MiaPaCa-2 cells, we examined the expression patterns of p53 family members and their related gene products under the same experimental conditions. In support of the above observations, siRNA-based silencing of mutant *p53* augmented SAHA-induced accumulation of γH2AX, cleaved PARP, TAp63 and p21^WAF1^ (Fig 5A). These observations indicate that TAp63 is implicated in SAHA-mediated cell death induction of MiaPaCa-2 cells. Indeed, knockdown of *p63* partially prohibited cell death following SAHA treatment (S3 Fig). Intriguingly, knockdown of mutant *p53* led to a marked decrease in the amount of RUNX2 at both protein and mRNA levels irrespective of SAHA treatment (Fig 5A and 5B), suggesting that RUNX2 might be one of mutant p53-target gene products and also participate in poor response to SAHA.

As shown in S4 Fig, mutant *p53* depletion resulted in a two-fold increase in sub-G1 cell population of Panc-1 cells following SAHA exposure. Similar to MiaPaCa-2 cells, SAHA-mediated further up-regulation of TAp63 and p21^WAF1^ was observed in mutant *p53*-knocked down Panc-1 cells in association with an obvious down-regulation of RUNX2 (S5 Fig). In contrast to MiaPaCa-2 cells, however, mutant *p53* gene silencing did not promote PARP cleavage in Panc-1 cells exposed to SAHA. Since mutant *p53* depletion in Panc-1 cells further augmented SAHA-induced decrease in cell viability and also increase in the amount of cell cycle-related p21^WAF1^, it is possible that, unlike MiaPaCa-2 cells, mutant *p53* knockdown in Panc-1 cells might contribute largely to the potentiation of SAHA-mediated cell cycle arrest/cell cycle retardation rather than cell death. Although it is speculated that Panc-1 cells retain SAHA-dependent pro-arrest pathway (TAp63-mediated and/or mutant *p53* depletion-stimulated up-regulation of p21^WAF1^), whereas SAHA-induced pro-apoptotic pathway is partially disrupted, the precise molecular mechanism(s) behind this phenomenon is unclear.

### Depletion of *RUNX2* enhances SAHA sensitivity and down-regulates mutant p53 expression

**Fig 6:**
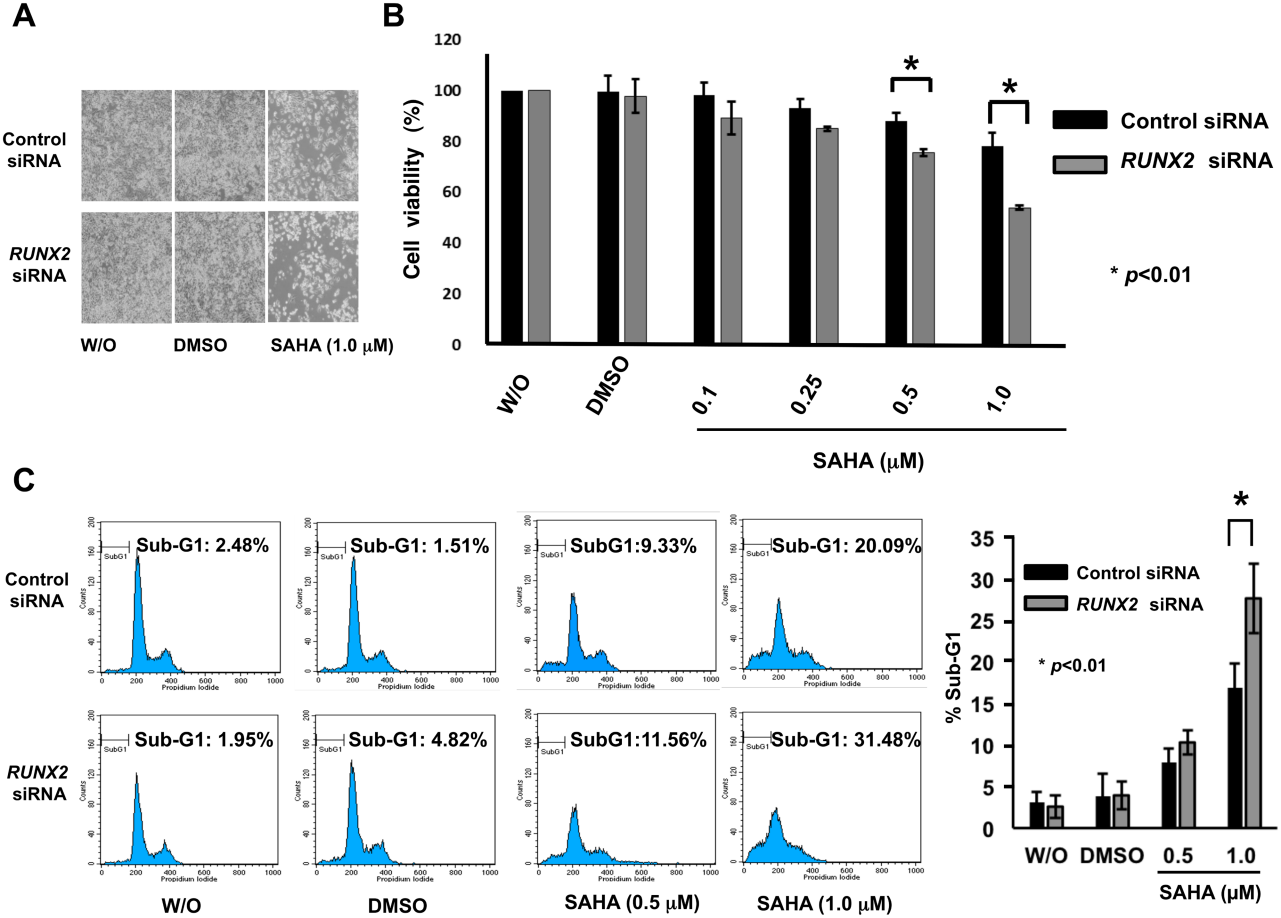
Knockdown of *RUNX2* potentiates SAHA-dependent cell death of MiaPaCa-2 cells. (A) Representative photos. MiaPaCa-2 cells were transfected with control siRNA or with siRNA targeting *RUNX2*. Twenty-four hours after transfection, cells were treated with DMSO, 1 μM of SAHA or left untreated. Forty-eight hours after treatment, the representative pictures were taken. (B) Cell survival assay. MiaPaCa-2 cells were transfected with control siRNA or with siRNA targeting *RUNX2*, and treated with DMSO or with the indicated concentrations of SAHA. Forty-eight hours after treatment, cells were analyzed by WST cell survival assay. Solid and grey boxes indicate control siRNA- and *RUNX2* siRNA-transfected cells, respectively. (C) FACS analysis. MiaPaCa-2 cells were transfected with control siRNA or with siRNA targeting *RUNX2*, and treated with DMSO or with the indicated concentrations of SAHA. Forty-eight hours after treatment, floating and adherent cells were harvested and subjected to flow cytometric analysis. Solid and grey boxes indicate control siRNA- and *RUNX2* siRNA-transfected cells, respectively.

To test the above hypothesis that, like mutant p53, RUNX2 could contribute to SAHA resistance of *p53*-mutated pancreatic cancer cells, we sought to assess the possible impact of *RUNX2* knockdown on SAHA-mediated cell death and mutant p53 expression in MiaPaCa-2 cells. For this purpose, we have checked knockdown efficiency of 3 different siRNAs against *RUNX2* (*RUNX2* siRNA-1, -2 and -3). Among them, *RUNX2* siRNA-3 showed the highest knockdown efficiency as examined by RT-PCR and immunoblotting (S6 Fig). Then, we utilized *RUNX2* siRNA-3 (termed *RUNX2* siRNA, thereafter) for further experiments. As seen in Fig 6A and 6B, *RUNX2* gene silencing caused a significant reduction in number of viable cells and also decreased cell viability following SAHA exposure. Similar to forced reduction of mutant *p53* mediated by siRNA, flow cytometric analysis revealed that SAHA-dependent increase in sub-G1 cell population is further augmented in *RUNX2*-depleted cells (Fig 6C).

**Fig 7:**
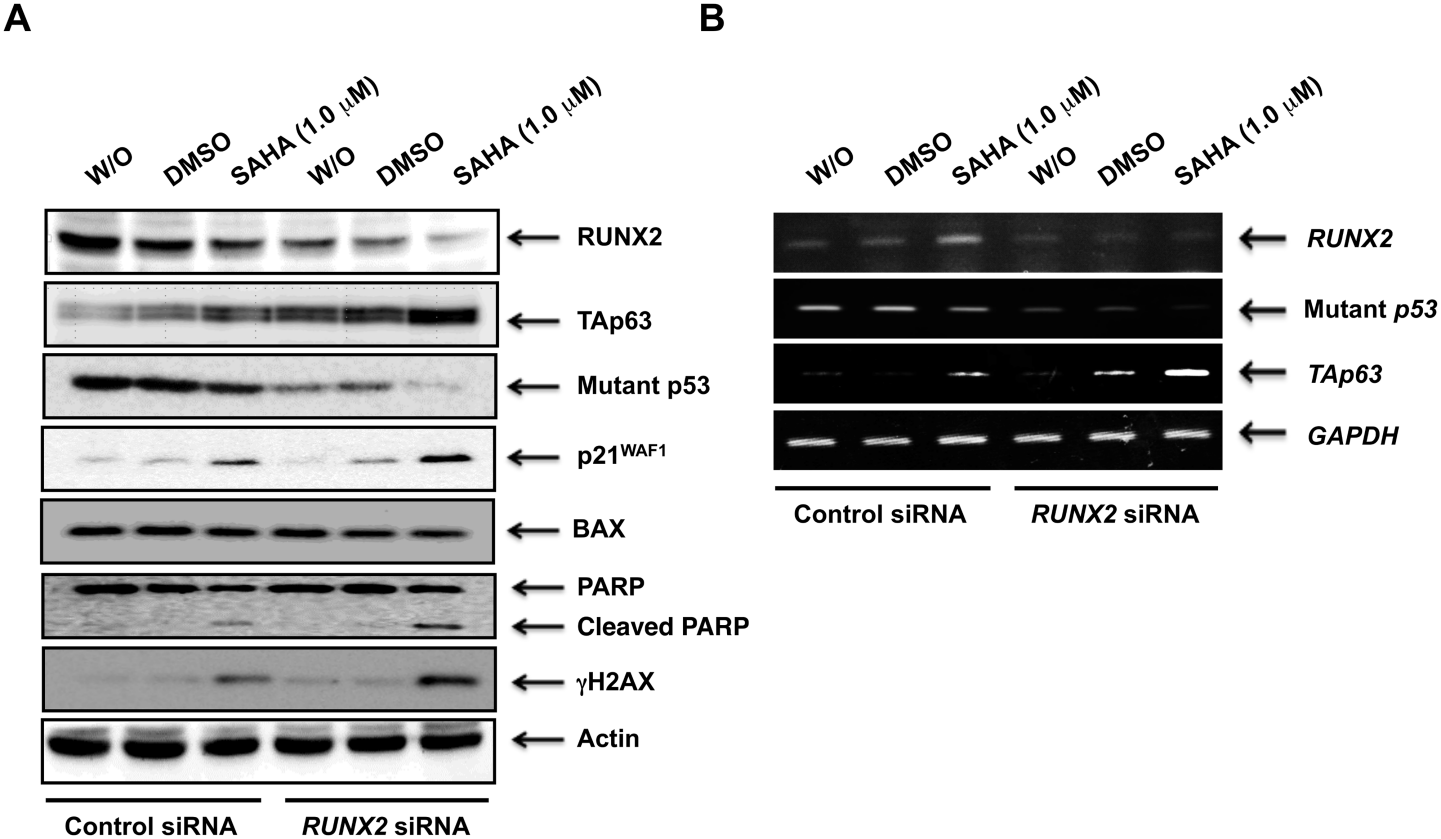
Silencing of *RUNX2* stimulates SAHA-induced accumulation of TAp63 and reduction of mutant p53. MiaPaCa-2 cells were transfected and treated as in Fig 6A. Forty-eight hours after treatment, cell lysates and total RNA were extracted and processed for immunoblotting (A) and RT-PCR (B), respectively. Actin and *GAPDH* were used as a loading and an internal control, respectively.

These observations prompted us to examine the expression patterns of p53 family members and their related gene products under the same experimental conditions. As seen in Fig 7A, SAHA-mediated up-regulation of γH2AX, cleaved PARP, TAp63 and p21^WAF1^ was further stimulated in *RUNX2*-knocked down cells. Unlike p21^WAF1^, BAX remained unchanged regardless of *RUNX2* knockdown. Of note, forced depletion of *RUNX2* caused a significant down-regulation of mutant p53 at both mRNA and protein levels (Fig 7A and 7B). Thus, it is possible that mutant p53 might be at least in part under the control of RUNX2.

### Forced expression of RUNX2 restores SAHA-dependent decrease in cell viability and increases the amount of mutant p53

**Fig 8:**
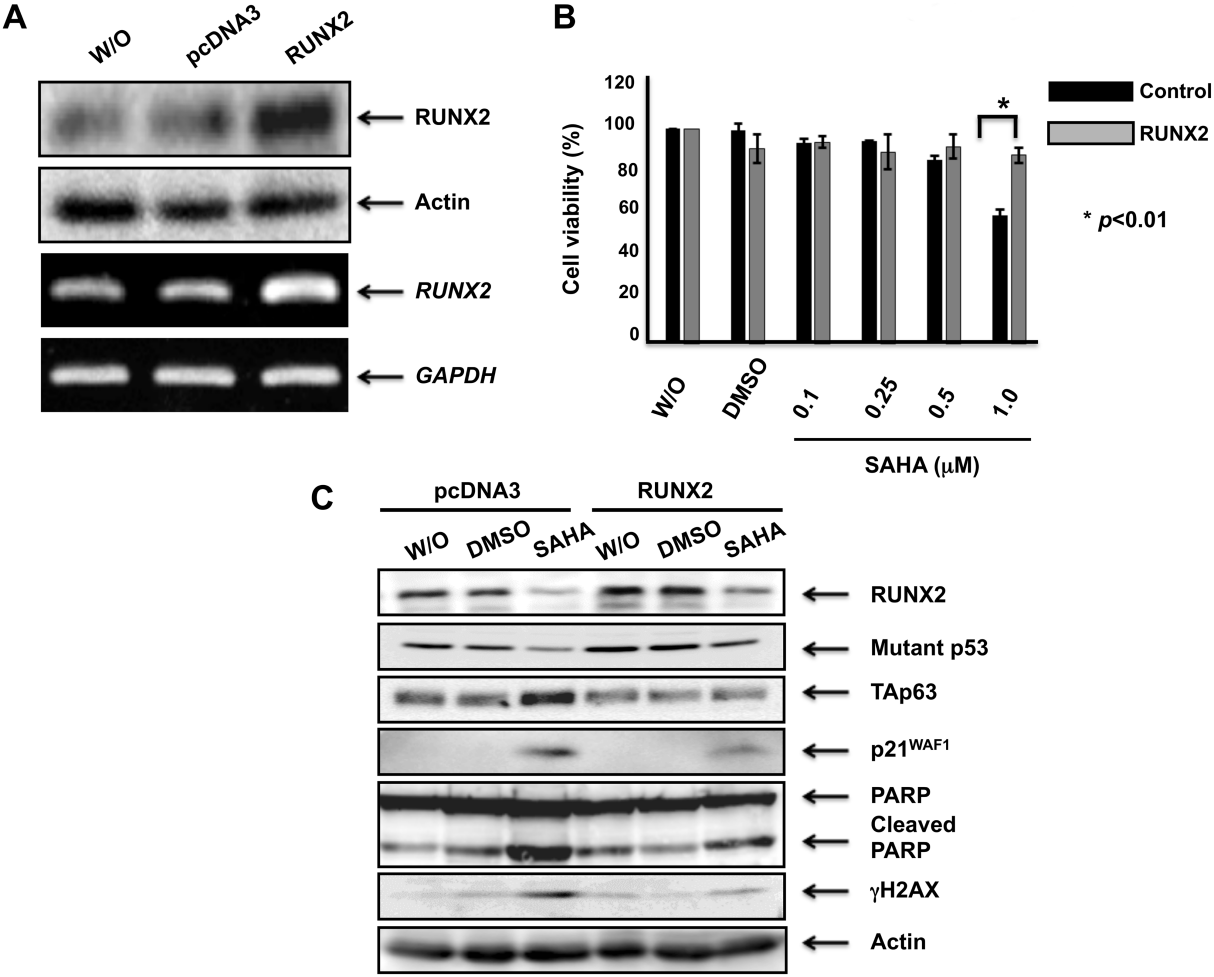
Forced expression of RUNX2 restores SAHA-mediated decrease in cell viability and further increases the amount of mutant p53. (A) Overexpression of RUNX2. MiaPaCa-2 cells were transfected with pcDNA3 or with the expression plasmid for RUNX2. Forty-eight hours after transfection, cell lysates and total RNA were prepared and subjected to immunoblotting (upper panels) and RT-PCR (lower panels), respectively. Actin and *GAPDH* were used as a loading and an internal control, respectively. (B) WST assay. MiaPaCa-2 cells were transfected as in (A). Twenty-four hours after transfection, cells were treated with DMSO, the indicated concentrations of SAHA or left untreated. Forty-eight hours after treatment, cell viability was examined by WST cell survival assay. (C) Immunoblotting. MiaPaCa-2 cells were transfected as in (A). Twenty-four hours after transfection, cells were treated with DMSO, 1 μM of SAHA or left untreated. Forty-eight hours ater treatment, cell lysates were extracted and analyzed by immunoblotting with the indicated antibodies. Actin was used as a loading control.

To confirm the results obtained from *RUNX2* gene silencing, MiaPaCa-2 cells were transfected with the empty plasmid or with the expression plasmid for RUNX2. As shown in Fig 8A, forced expression of RUNX2 was successful under our experimental conditions. After transfection, cells were treated with DMSO, the indicated concentrations of SAHA or left untreated. Forty-eight hours post-treatment, cell viability was assessed by WST cell survival assay. As seen in Fig 8B, SAHA-dependent decrease in cell viability was obviously restored by overexpression of RUNX2 (1 μM of SAHA exposure). Under the same experimental conditions, cell lysates were prepared and analyzed by immunoblotting. Consistent with the results obtained from WST assay, SAHA-induced accumulation of γH2AX as well as cleaved PARP was attenuated by forced expression of RUNX2 (Fig 8C). As expeced, overexpression of RUNX2 increased the amount of mutant p53 and suppressed p21^WAF1^ as well as TAp63 expression in response to SAHA. Given that depletion of mutant *p53* and *RUNX2* reduces the expression level of RUNX2 and mutant p53, respectively, it is likely that there exists a positive auto-regulatory feedback loop between mutant p53 and RUNX2, and this regulatory system might contribute to the low sensitivity of MiaPaCa-2 cells against SAHA.

## Discussion

SAHA, which is a prototype of the newly developed HDAC inhibitor with a low toxicity, markedly elevated the acetylation level of histone H3/H4, and also lowered the expression level of various HDACs such as HDAC1, 2, 4 and 7 [38]. Unfortunately, it has become evident that, unlike T-cell lymphoma, certain solid tumors including pancreatic, colon, prostate and non-small cell lung cancers, sometimes poorly respond to SAHA [39, 40]. To improve the efficacy of SAHA on these tumors, it is urgent to precisely understand the molecular mechanism(s) behind this low sensitivity to SAHA of certain solid tumor cells. In the current study, we have found for the first time that SAHA-induced cell death of *p53*-mutated pancreatic cancer MiaPaCa-2 cells is modulated at least in part through RUNX2/mutant p53/TAp63-regulatory axis.

According to our present observations, there existed an inverse relationship between the expression levels of RUNX2 and TAp63 in MiaPaCa-2 cells in response to SAHA. Upon SAHA exposure, TAp63 and RUNX2 were up- and down-regulated at protein level, respectively. Depletion of *RUNX2* enhanced cell death response evoked by SAHA accompanied by a further stimulation of TAp63 expression, indicating that RUNX2 negatively regulates TAp63 expression. Forced expression of RUNX2 restored SAHA-mediated decrease in cell viability and lowered the amount of TAp63. Recently, we have demonstrated that RUNX2 prohibits GEM-mediated cell death of *p53*-null pancreatic cancer AsPC-1 cells as well as *p53*-mutated pancreatic cancer Panc-1 cells through down-regulation of TAp63-dependent cell death pathway [24, 41]. Thus, It is suggestive that TAp63 plays a vital role in the regulation of DNA damage-mediated cell death of pancreatic cancer cells without functional p53. Indeed, silencing of *p63* partially attenuated SAHA-induced cell death of MiaPaCa-2 cells. In support of our present findings, Shim *et al*. described that the ectopic expression of TAp63 augments SAHA-mediated cell death of head and neck squamous cell carcinoma [42].

As described [21, 22], in addition to pro-osteogenic target genes, RUNX2 has an ability to transactivate a variety of its target genes implicated in angiogenesis, invasiveness and/or metastasis such as *Vegf*, *Spp1*, *MMP9* and *MMP13*. Shin *et al*. found that MYC is a novel mediator of pro-survival function of RUNX2 [43]. Based on their results, depletion of *RUNX2* in osteosarcoma cells remarkably stimulated cell death irrespective of *p53* status, and RUNX2 was capable to transactivate pro-survival c-*myc*. In a good agreement with these results, forced expression of MYC prohibited cell death caused by *RUNX2* gene silencing. Thus, their observations strongly suggest that RUNX2 might exert its pro-survival/pro-oncogenic function at least in part through the induction of MYC expression. It is of interest to examine whether this pro-oncogenic RUNX2/MYC-regulatory axis could also participate in the determination of SAHA sensitivity of *p53*-mutated pancreatic cancer cells. Additional studies should be required to address this issue.

Wang *et al*. described that SAHA causes a massive reduction of mutant p53 through the disruption of the physical interaction between YY1 and HDAC8 [44]. According to their findings, YY1 bound to the promoter region of mutant *p53* (at position from -102 to -96 relative to the transcription initiation site) and induced its transcription. Similar to their observations, we have found that the amount of mutant p53 expressed in MiaPaCa-2 cells is significantly lowered in response to SAHA both at mRNA and protein levels. SAHA-mediated down-regulation of mutant p53 was accompanied by a marked reduction of RUNX2. Of note, knockdown of *RUNX2* led to an obvious decrease in mutant p53 both at mRNA and protein levels, suggesting that RUNX2 might positively regulate mutant *p53* transcription. Alternatively, Li *et al*. described that SAHA facilitates the release of mutant p53 from HSP90/HDAC6 complex and thereby promoting its degradation mediated by MDM2 and CHIP in human breast cancer-derived MDA231 cells [45]. From their results, HDAC6 which acts as a positive regulator of HSP90 chaperone machinery, was responsible for the hyperstability of mutant p53. Since we have revealed that RUNX2 forms a complex with HDAC6 in cells [23], it is likely that RUNX2 functions as a scaffold protein to keep HSP90/HDAC6 complex stable, and thus protects mutant p53 from MDM2/CHIP-mediated degradation. Further studies should be required to address whether SAHA-mediated degradation of mutant p53 could also take place in pancreatic cancer cells.

Intriguingly, depletion of mutant *p53* led to a massive reduction in RUNX2 both at mRNA and protein levels. From our present results, mutant *p53*-knocked down MiaPaCa-2 cells underwent cell death much more efficiently in response to SAHA (approximately 2-fold increase) relative to non-silencing control cells exposed to SAHA. A growing body of evidence strongly indicates that the acquired pro-oncogenic function of mutant p53 is largely attributed to the direct and/or indirect ability to regulate its target gene expression [46]. For example, mutant p53 drives the expression of PML, which is responsible for pro-oncogenic activity of mutant p53 [47]. Frazier *et al*. described that pro-oncogenic c-*myc* is a direct transcriptional target of mutant p53 [48]. In addition, Sampath *et al*. demonstrated that mutant p53 transactivates *MDR* (multidrug resistance) but not *MRP1* (multidrug resistance-associated protein 1) [49]. Recently, it has been shown that mutant p53 enhances etoposide resistance through ETS2-mediated up-regulation of *TDP2* (5′-tyrosyl DNA phosphodiesterase) [50]. Since knockdown of mutant *p53* down-regulated RUNX2 both at mRNA and protein levels, it is suggestive that *RUNX2* might be one of the direct or indirect transcriptional target genes of mutant p53. Alternatively, RUNX2 might positively regulate mutant p53 expression as described above. Together, it is possible that there exists a positive feedback regulatory loop between RUNX2 and mutant p53, and thereby amplifing the signal(s) essential for the low sensitivity to SAHA of MiaPaCa-2 cells. Similarly, Suenaga *et al*. found that the positive feedback regulation between oncogenic MYCN and NCYM contributes to the malignant phenotype of MYCN/NCYM-amplified neuroblastomas [51].

Although TAp63 was induced following SAHA exposure, its transactivation-mediated pro-apoptotic actvity might be impeded by mutant p53 which acts as a dominant-negative inhibitor against TAp63 [12]. Recently, we have shown that RUNX2 impairs the transcriptional activity of TAp63 through the complex formation [41]. Based on our present observations, silencing of *RUNX2* significantly augmented SAHA-dependent induction of TAp63 and also massively down-regulated mutant p53, leading to an enhancement of SAHA-mediated cell death. These observations implys that TAp63 escapes from its negative regulator RUNX2 as well as mutant p53, exerts its pro-apoptotic activity, and thereby improving SAHA sensitivity of MiaPaCa-2 cells. Wei *et al*. demonstrated that ATF3 (activating transcription factor 3) whose expression is suppressed by mutant p53, disrupts the interaction between mutant p53 and TAp63, and thus sensitizes tumor cells bearing *p53* mutation to anti-cancer drugs [52]. Moreover, ATF3 was associated with p53 mutants and prohibited their pro-oncogenic function. From their results, ATF3 bound to the hot spot p53 mutants such as R175H and R273H, and then attenuated their pro-oncogenic activities. MiaPaCa-2 cells express p53 mutants (R273H/R248W), suggesting that R273H mutant expressed in MiaPaCa-2 cells might be at least in part suppressed by ATF3. With this in mind, it is conceivable that ATF3 enhances SAHA sensitivity of MiaPaCa-2 cells through the inhibition of mutant p53 (R273H) and/or reactivation of TAp63. Of note, Gokulnath *et al*. provided the evidence showing that ATF3 is recruited onto the promoter region of *RUNX2* in breast cancer cells [53]. Although their findings suggest the presence of functional interaction between ATF3 and RUNX2, further studies should be necessary to clarify whether ATF3 could be involved in RUNX2/mutant p53/TAp63-regulatory axis in response to SAHA.

It is well known that the induction of DNA double-strand breaks (DSBs) leads to the rapid phosphorylation of chromatin H2AX at Ser-139 (γH2AX), which is catalyzed by ATM (Ataxia telangiectasia mutated), ATR (ataxia telangiectasia- and Rad3-related kinase) and/or DNA-PK (DNA-dependent protein kinase). Since DNA damage-mediated generation of γH2AX expands over a large chromatin domain flanking DSBs, the accumulation of γH2AX is generally regarded as DSB marker [54]. Another finding of our present study was that forced depletion and expression of *RUNX2* increases and decreases SAHA-mediated accumulation of γH2AX, respectively. Similar to *RUNX2* gene silencing, knockdown of mutant *p53* resulted in a significant increase in the amount of γH2AX in response to SAHA. Recently, we have described that *RUNX2* gene silencing augments GEM-induced accumulation of γH2AX in MiaPaCa-2 cells [26]. These results indicate that RUNX2 might participate in the regulation of DNA damage-dependent phosphorylation of H2AX.

In summary, we have found for the first time that the positive auto-regulatory loop between RUNX2 and mutant p53 contributes to poor response to SAHA of *p53*-mutated pancreatic cancer MiaPaCa-2 cells through the down-regulation of TAp63. Thus, our present findings strongly suggest that the disruption of this positive auto-regulatory system might provide a clue to develop a novel strategy to treat advanced pancreatic cancer patients harboring *p53* mutation.

## Supporting information

S1 Fig *p53*-mutated human pancreatic cancer MiaPaCa-2 and Panc-1 cells respond poorly to SAHA. MiaPaCa-2 (A) and Panc-1 (B) cells were exposed to DMSO, the indicated concentrations of SAHA or left untreated (W/O). At the indicated time periods after treatment, floating and attached cells were harvested and their DNA content was examined by flow cytometric analysis.
(PPT)

S2 Fig Inverse relationship between the expression levels of mutant p53/RUNX2 and TAp63 in Panc-1 cells following SAHA exposure. Panc-1 cells were treated with DMSO, the increasing amounts of SAHA or left untreated. Forty-eight hours after treatment, cell lysates and total RNA were prepared and subjected to immunoblotting (A) and RT-PCR (B), respectively. Actin and *GAPDH* were used as a loading and an internal control, respectively.
(PPT)

S3 Fig Knockdown of *p63* partially attenuates SAHA-induced cell death of MiaPaCa-2 cells. (A) siRNA-mediated knockdown of *p63*. MiaPaCa-2 cells were transfected with control siRNA or with the indicated siRNAs against *p63* (*p63* siRNA-1, *p63* siRNA-2, and *p63* siRNA-3). Twenty-four hours after transfection, total RNA and cell lysates were isolated and analyzed by RT-PCR (upper panels) and immunoblotting (lower panels), respectively. *GAPDH* and actin were used as an internal and a loading control, respectively. (B) FACS analysis. MiaPaCa-2 cells were transfected with control siRNA or with *p63* siRNA (*p63* siRNA-2), and then treated with DMSO, SAHA (0.5 μM or 1 μM) or left untreated. Forty-eight hours after treatment, floating and attached cells were harvested and subjected to flow cytometric analysis. Solid and grey boxes indicate control siRNA- and *p63* siRNA-transfected cells, respectively.
(PPT)

S4 Fig Silencing of mutant *p53* in Panc-1 cells stimulates SAHA-dependent decrease and increase in cell viability and cell death, respectively. (A) Phase-contrast micrographs. Panc-1 cells were transfected with control siRNA or with siRNA against *p53*, and then treated with DMSO, 1 μM of SAHA or left untreated. Forty-eight hours after treatment, the representative pictures were taken. (B) WST assay. Panc-1 cells were transfected and treated with DMSO or with the indicated concentrations of SAHA. Forty-eight hours after SAHA exposure, cells were analyzed by the standard WST cell survival assay. Solid and grey boxes indicate control siRNA- and *p53* siRNA-transfected cells, respectively. (C) FACS analysis. Panc-1 cells were transfected and treated with DMSO or with 1 μM of SAHA. Forty-eight hours after treatment, floating and adherent cells were harvested and subjected to flow cytometric analysis. Solid and grey boxes indicate control siRNA- and *p53* siRNA-transfected cells, respectively.
(PPT)

S5 Fig Forced depletion of mutant *p53* augments SAHA-mediated accumulation of TAp63 and reduction of RUNX2. Panc-1 cells were transfected and treated as in S4A Fig. Forty-eight hours post-treatment, cell lysates and total RNA were prepared and analyzed by immunoblotting (A) and RT-PCR (B), respectively. Actin and *GAPDH* were used as a loading and an internal control, respectively.
(PPT)

S6 Fig siRNA-mediated knockdown of *RUNX2*. MiaPaCa-2 cells were transfected with control siRNA or with the indicated siRNAs against *RUNX2* (*RUNX2* siRNA-1, *RUNX2* siRNA-2, and *RUNX2* siRNA-3). Forty-eight hours after transfection, total RNA and cell lysates were prepared and analyzed by RT-PCR (upper panels) and immunoblotting (lower panels), respectively. *GAPDH* and actin were used as an internal and a loading control, respectively.
(PPT)

## Abbreviations

GAPDH: glyceraldehyde 3-phosphate dehydrogenase
GEM: gemcitabine
KD: knockdown
PARP: Poly (ADP-ribose) polymerase
PBS: phosphate-buffered saline
PCR: polymerase chain reaction
PI: propidium iodide
RT: reverse transcription
RUNX2: runt-related transcription factor 2
SAHA: suberoylanilide hydroxamic acid
TBS: Tris-buffered saline
