# Supplementary material for: Depletion of runt-related transcription factor 2 (RUNX2) enhances SAHA sensitivity of p53-mutated pancreatic cancer cells through the regulation of mutant p53 and TAp63
Source: PLoS One. 2017 Jul 3;12(7):e0179884. doi: 10.1371/journal.pone.0179884 (PMC5495219; doi:10.1371/journal.pone.0179884)
Supplement: S1 Fig — MiaPaCa-2 (A) and Panc-1 (B) cells were exposed to DMSO, the indicated concentrations of SAHA or left untreated (W/O). At the indicated time periods after treatment, floating and attached cells were harvested and their DNA content was examined by flow cytometric analysis. (PPT) [file pone.0179884.s001.ppt]

## Slide 1
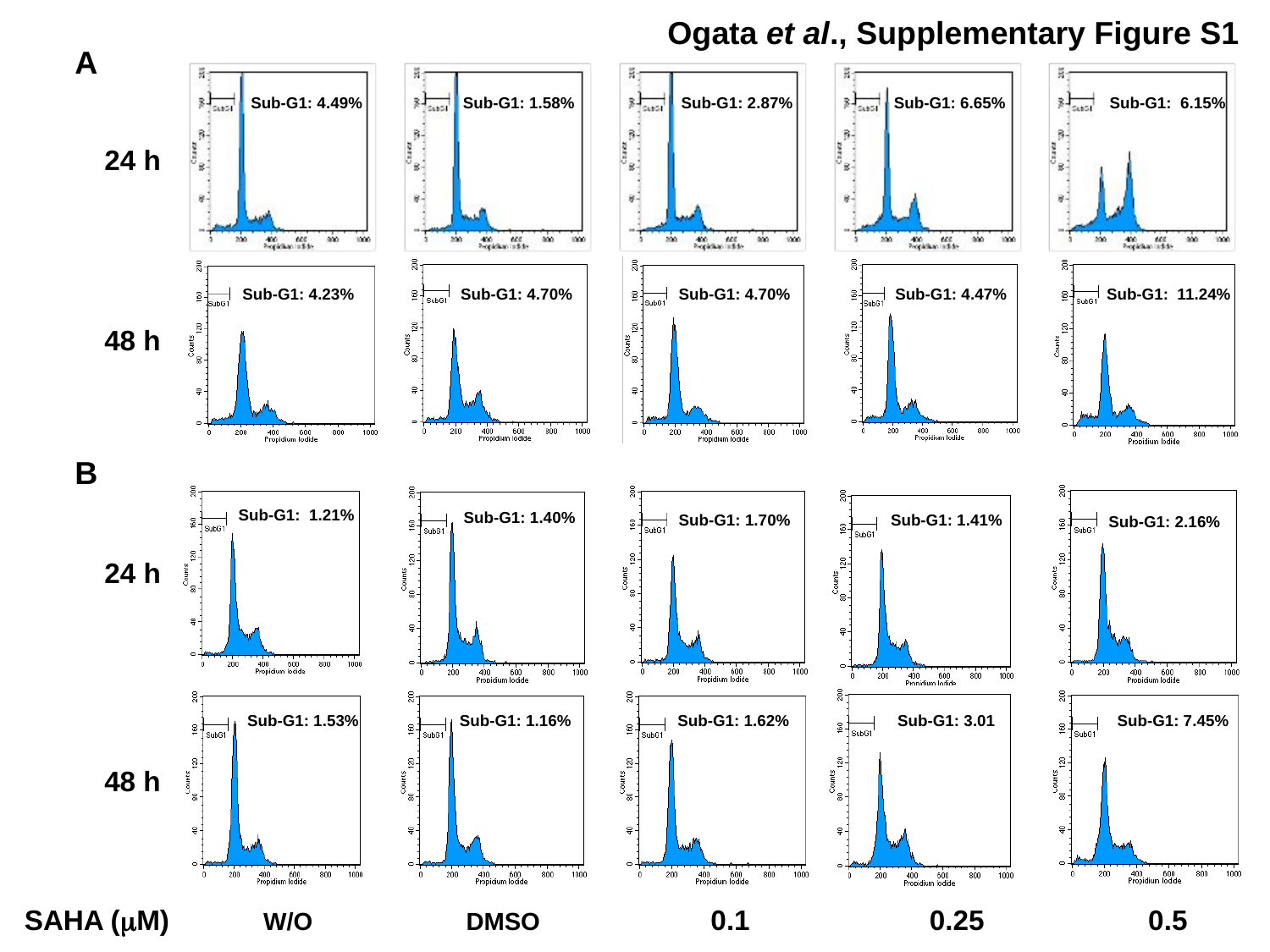

Ogata et al., Supplementary Figure S1
A
Sub-G1: 4.49%
Sub-G1: 1.58%
Sub-G1: 2.87%
Sub-G1: 6.65%
Sub-G1: 6.15%
24 h
Sub-G1: 4.23%
Sub-G1: 4.70%
Sub-G1: 4.70%
Sub-G1: 4.47%
Sub-G1: 11.24%
48 h
B
Sub-G1: 1.21%
Sub-G1: 1.40%
Sub-G1: 1.70%
Sub-G1: 1.41%
Sub-G1: 2.16%
24 h
Sub-G1: 1.53%
Sub-G1: 1.16%
Sub-G1: 1.62%
Sub-G1: 3.01
Sub-G1: 7.45%
48 h
SAHA (μM) W/O DMSO 0.1 0.25 0.5
