# Supplementary material for: Depletion of runt-related transcription factor 2 (RUNX2) enhances SAHA sensitivity of p53-mutated pancreatic cancer cells through the regulation of mutant p53 and TAp63
Source: PLoS One. 2017 Jul 3;12(7):e0179884. doi: 10.1371/journal.pone.0179884 (PMC5495219; doi:10.1371/journal.pone.0179884)
Supplement: S2 Fig — Panc-1 cells were treated with DMSO, the increasing amounts of SAHA or left untreated. Forty-eight hours after treatment, cell lysates and total RNA were prepared and subjected to immunoblotting (A) and RT-PCR (B), respectively. Actin and GAPDH were used as a loading and an internal control, respectively. (PPT) [file pone.0179884.s002.ppt]

## Slide 1
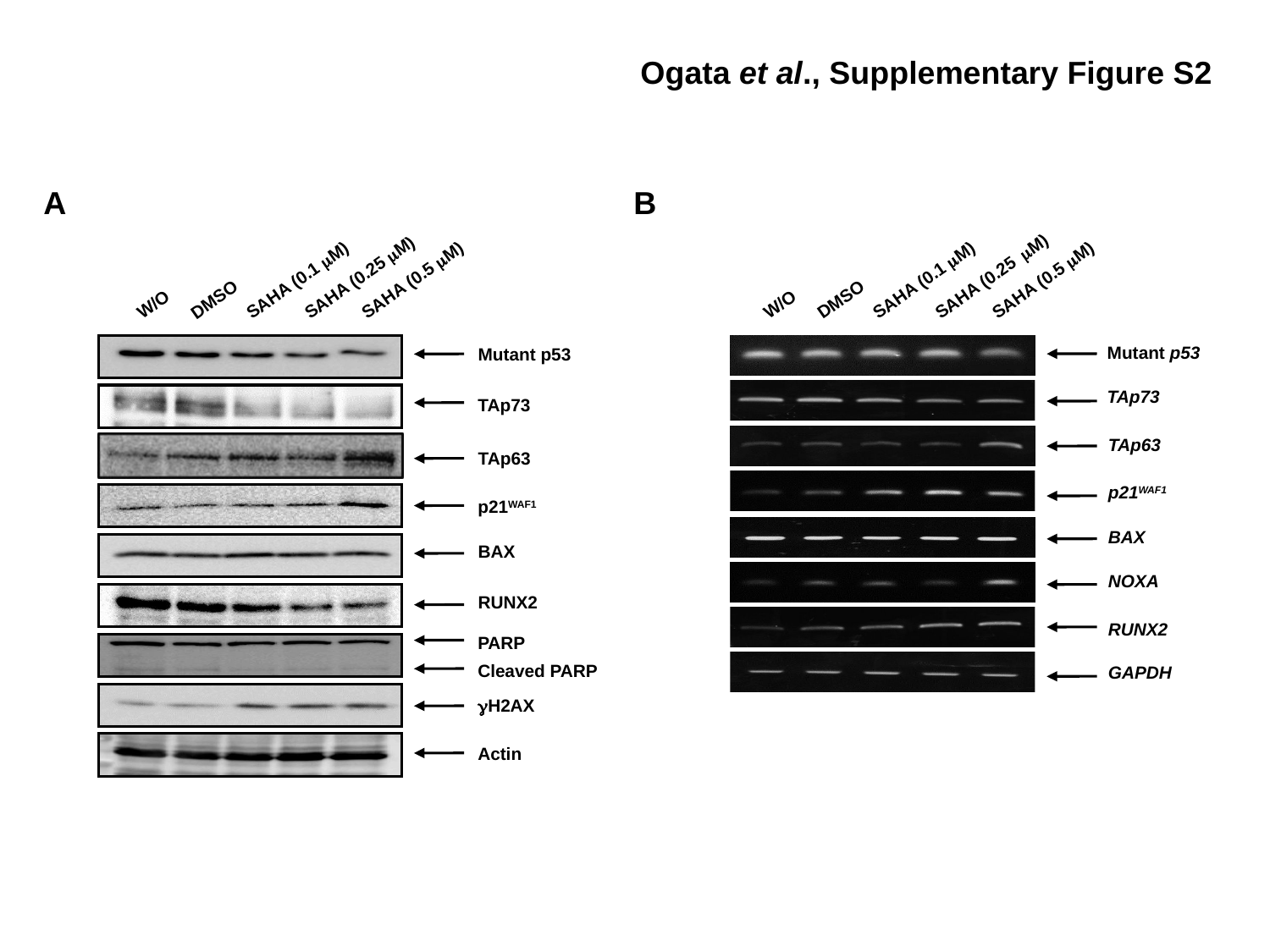

Ogata et al., Supplementary Figure S2
A
B
SAHA (0.5 μM)
SAHA (0.1 μM)
SAHA (0.25 μM)
DMSO
W/O
SAHA (0.5 μM)
SAHA (0.25 μM)
SAHA (0.1 μM)
DMSO
W/O
Mutant p53
Mutant p53
TAp73
TAp73
TAp63
TAp63
p21WAF1
p21WAF1
BAX
BAX
NOXA
RUNX2
RUNX2
PARP
Cleaved PARP
GAPDH
γH2AX
Actin
