# Supplementary material for: Depletion of runt-related transcription factor 2 (RUNX2) enhances SAHA sensitivity of p53-mutated pancreatic cancer cells through the regulation of mutant p53 and TAp63
Source: PLoS One. 2017 Jul 3;12(7):e0179884. doi: 10.1371/journal.pone.0179884 (PMC5495219; doi:10.1371/journal.pone.0179884)
Supplement: S3 Fig — (A) siRNA-mediated knockdown of p63. MiaPaCa-2 cells were transfected with control siRNA or with the indicated siRNAs against p63 (p63 siRNA-1, p63 siRNA-2, and p63 siRNA-3). Twenty-four hours after transfection, total RNA and cell lysates were isolated and analyzed by RT-PCR (upper panels) and immunoblotting (lower panels), respectively. GAPDH and actin were used as an internal and a loading control, respectively. (B) FACS analysis. MiaPaCa-2 cells were transfected with control siRNA or with p63 siRNA (p63 siRNA-2), and then treated with DMSO, SAHA (0.5 μM or 1 μM) or left untreated. Forty-eight hours after treatment, floating and attached cells were harvested and subjected to flow cytometric analysis. Solid and grey boxes indicate control siRNA- and p63 siRNA-transfected cells, respectively. (PPT) [file pone.0179884.s003.ppt]

## Slide 1
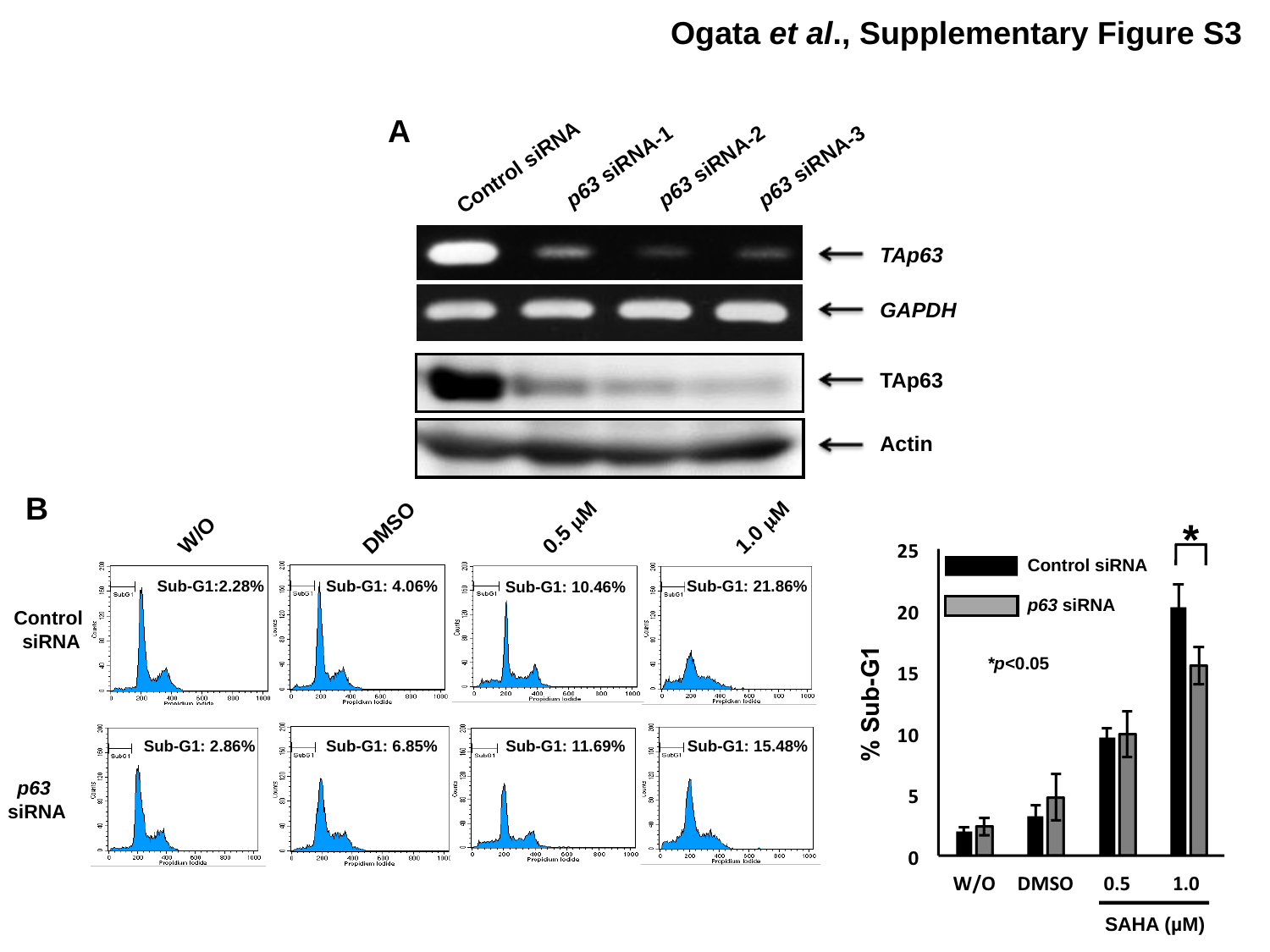

Ogata et al., Supplementary Figure S3
A
p63 siRNA-3
Control siRNA
p63 siRNA-1
p63 siRNA-2
TAp63
GAPDH
TAp63
Actin
B
DMSO
0.5 μM
1.0 μM
W/O
Sub-G1: 4.06%
Sub-G1: 21.86%
Sub-G1:2.28%
Sub-G1: 10.46%
Control
siRNA
Sub-G1: 2.86%
Sub-G1: 6.85%
Sub-G1: 11.69%
Sub-G1: 15.48%
p63
siRNA
*
Control siRNA
p63 siRNA
*p<0.05
SAHA (µM)
