# Supplementary material for: Depletion of runt-related transcription factor 2 (RUNX2) enhances SAHA sensitivity of p53-mutated pancreatic cancer cells through the regulation of mutant p53 and TAp63
Source: PLoS One. 2017 Jul 3;12(7):e0179884. doi: 10.1371/journal.pone.0179884 (PMC5495219; doi:10.1371/journal.pone.0179884)
Supplement: S4 Fig — (A) Phase-contrast micrographs. Panc-1 cells were transfected with control siRNA or with siRNA against p53, and then treated with DMSO, 1 μM of SAHA or left untreated. Forty-eight hours after treatment, the representative pictures were taken. (B) WST assay. Panc-1 cells were transfected and treated with DMSO or with the indicated concentrations of SAHA. Forty-eight hours after SAHA exposure, cells were analyzed by the standard WST cell survival assay. Solid and grey boxes indicate control siRNA- and p53 siRNA-transfected cells, respectively. (C) FACS analysis. Panc-1 cells were transfected and treated with DMSO or with 1 μM of SAHA. Forty-eight hours after treatment, floating and adherent cells were harvested and subjected to flow cytometric analysis. Solid and grey boxes indicate control siRNA- and p53 siRNA-transfected cells, respectively. (PPT) [file pone.0179884.s004.ppt]

## Slide 1
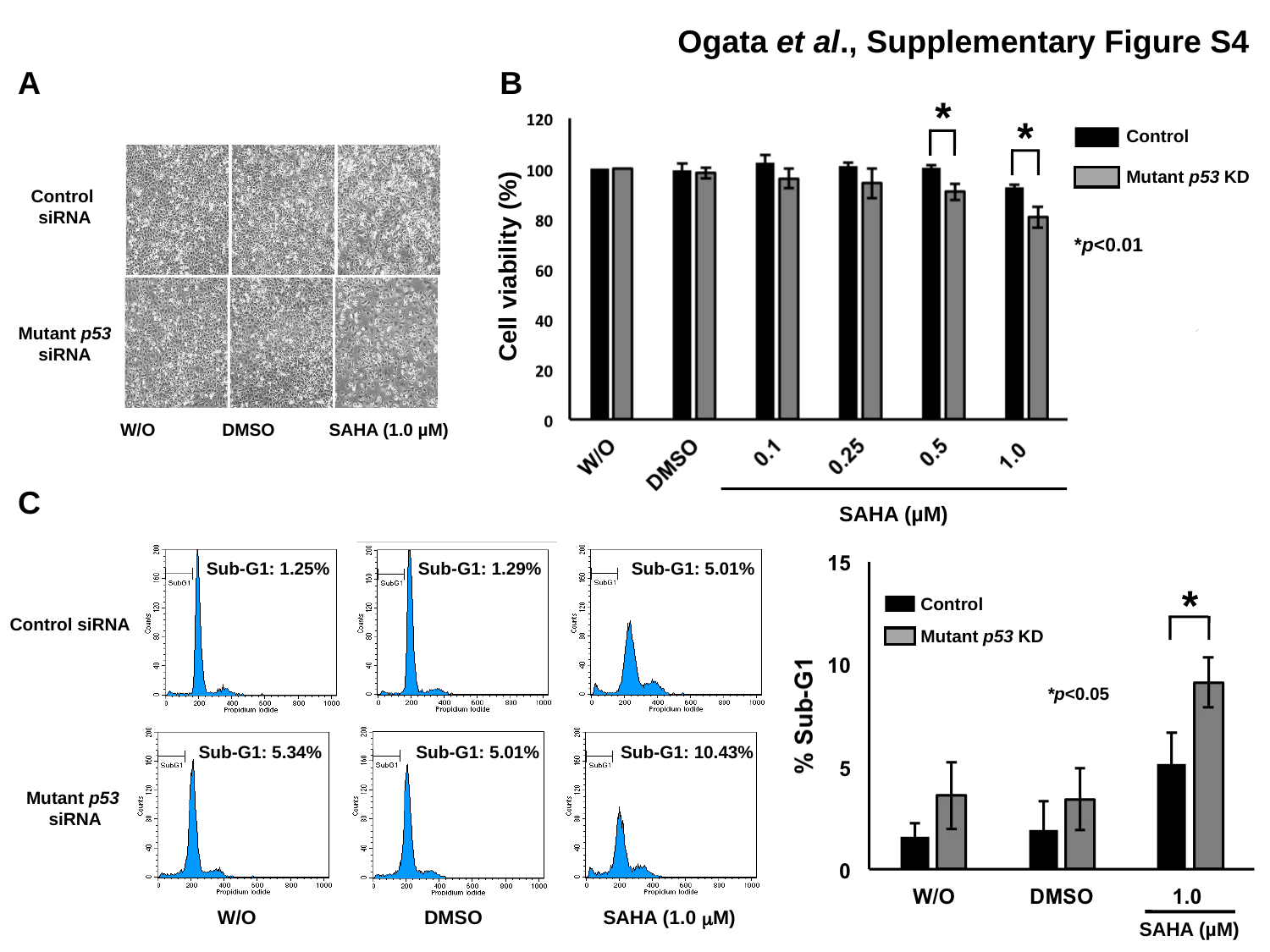

Ogata et al., Supplementary Figure S4
A
B
*
*
Control
Mutant p53 KD
Control
siRNA
Mutant p53
siRNA
W/O
DMSO
SAHA (1.0 µM)
 *p<0.01
Cell viability (%)
C
SAHA (µM)
Sub-G1: 1.25%
Sub-G1: 1.29%
Sub-G1: 5.01%
*
Control
Mutant p53 KD
Control siRNA
*p<0.05
Sub-G1: 5.34%
Sub-G1: 5.01%
Sub-G1: 10.43%
Mutant p53
siRNA
W/O
DMSO
SAHA (1.0 μM)
SAHA (µM)
