# Supplementary material for: Depletion of runt-related transcription factor 2 (RUNX2) enhances SAHA sensitivity of p53-mutated pancreatic cancer cells through the regulation of mutant p53 and TAp63
Source: PLoS One. 2017 Jul 3;12(7):e0179884. doi: 10.1371/journal.pone.0179884 (PMC5495219; doi:10.1371/journal.pone.0179884)
Supplement: S5 Fig — Panc-1 cells were transfected and treated as in S4A Fig. Forty-eight hours post-treatment, cell lysates and total RNA were prepared and analyzed by immunoblotting (A) and RT-PCR (B), respectively. Actin and GAPDH were used as a loading and an internal control, respectively. (PPT) [file pone.0179884.s005.ppt]

## Slide 1
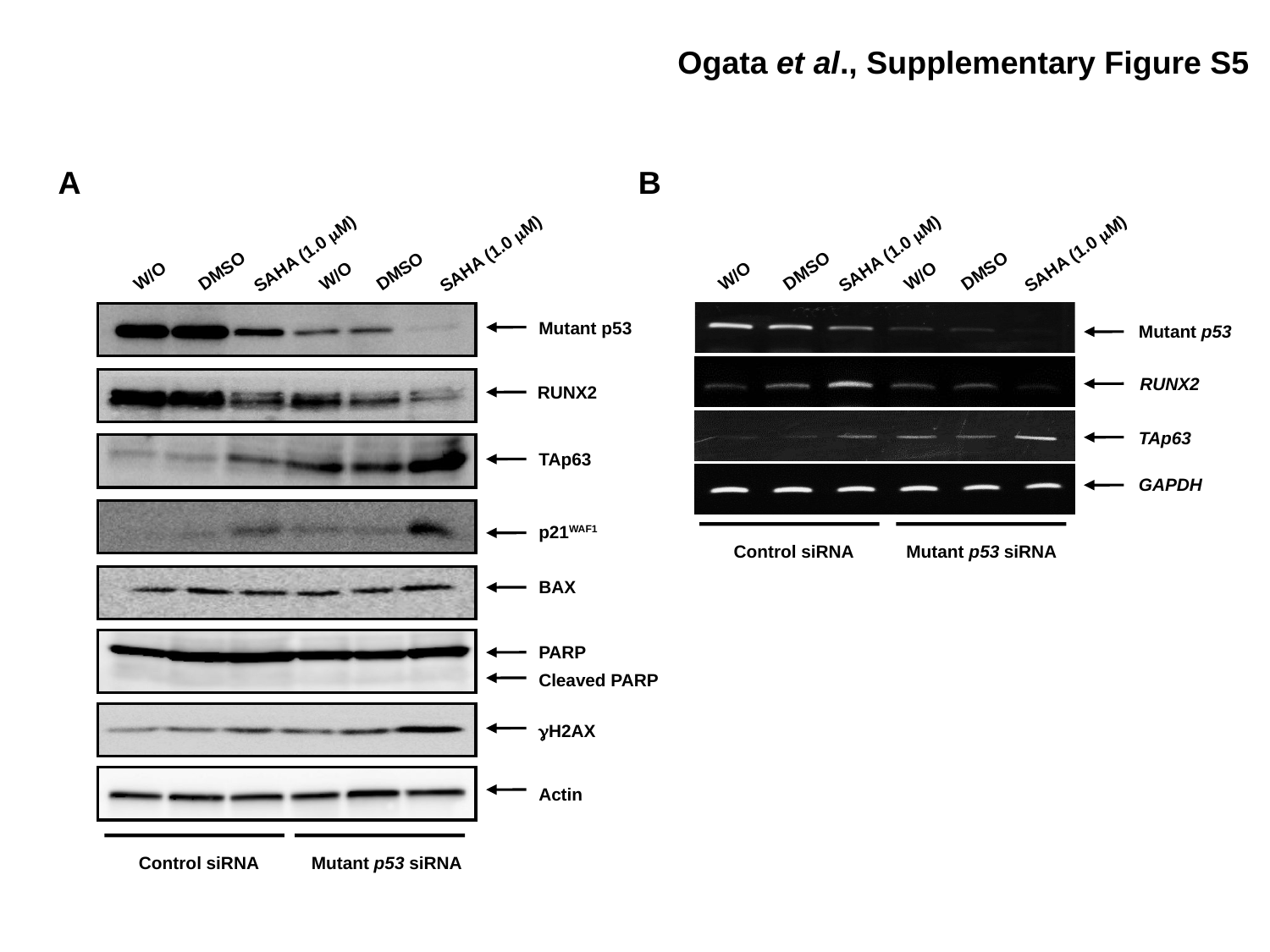

Ogata et al., Supplementary Figure S5
A
B
SAHA (1.0 μM)
SAHA (1.0 μM)
DMSO
DMSO
W/O
W/O
SAHA (1.0 μM)
SAHA (1.0 μM)
DMSO
DMSO
W/O
W/O
Mutant p53
Mutant p53
RUNX2
RUNX2
TAp63
TAp63
GAPDH
p21WAF1
Control siRNA
Mutant p53 siRNA
BAX
PARP
Cleaved PARP
γH2AX
Actin
Control siRNA
Mutant p53 siRNA
