# Supplementary material for: Depletion of runt-related transcription factor 2 (RUNX2) enhances SAHA sensitivity of p53-mutated pancreatic cancer cells through the regulation of mutant p53 and TAp63
Source: PLoS One. 2017 Jul 3;12(7):e0179884. doi: 10.1371/journal.pone.0179884 (PMC5495219; doi:10.1371/journal.pone.0179884)
Supplement: S6 Fig — MiaPaCa-2 cells were transfected with control siRNA or with the indicated siRNAs against RUNX2 (RUNX2 siRNA-1, RUNX2 siRNA-2, and RUNX2 siRNA-3). Forty-eight hours after transfection, total RNA and cell lysates were prepared and analyzed by RT-PCR (upper panels) and immunoblotting (lower panels), respectively. GAPDH and actin were used as an internal and a loading control, respectively. (PPT) [file pone.0179884.s006.ppt]

## Slide 1
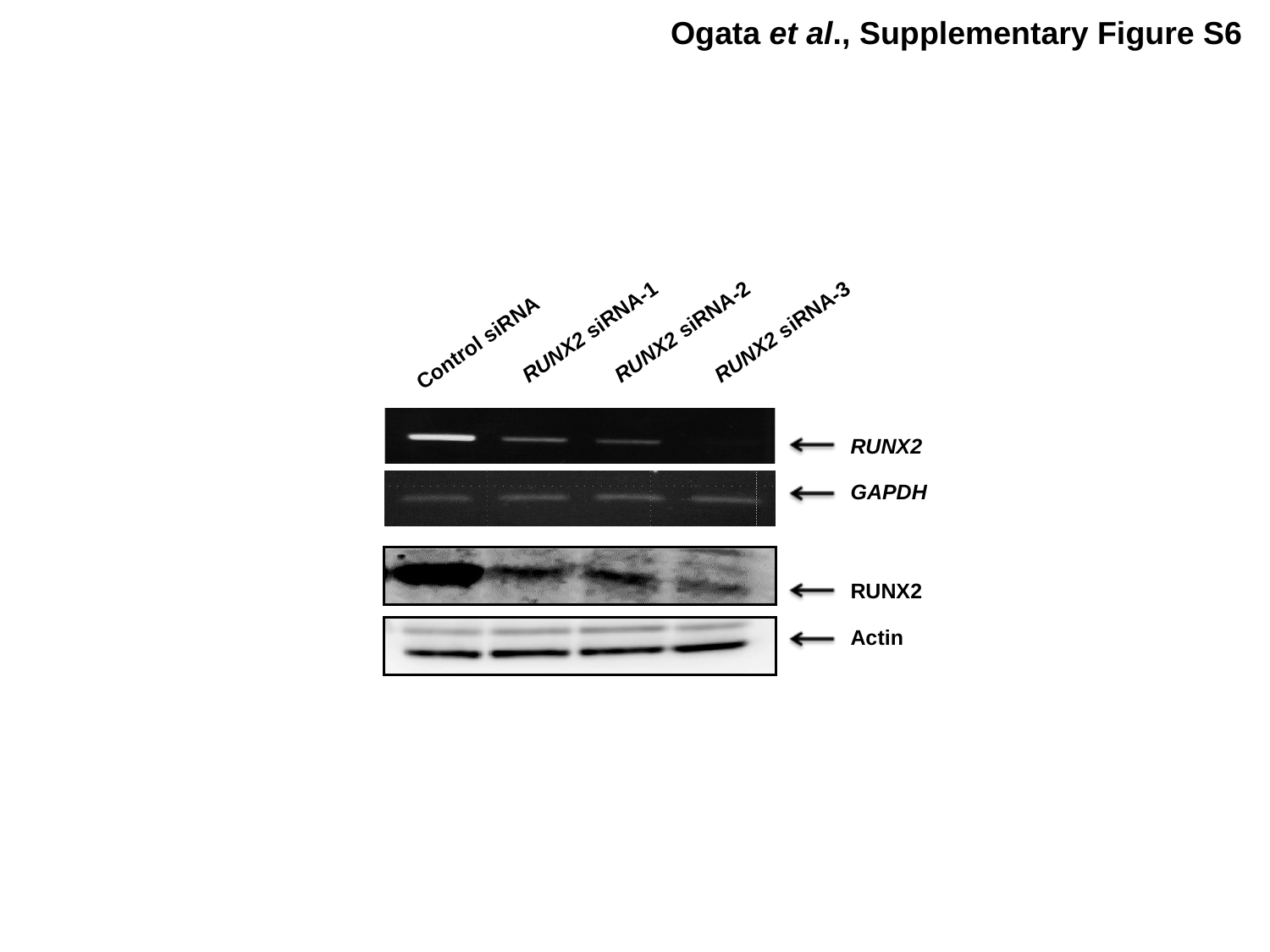

Ogata et al., Supplementary Figure S6
RUNX2 siRNA-3
Control siRNA
RUNX2 siRNA-1
RUNX2 siRNA-2
RUNX2
GAPDH
RUNX2
Actin
